# Cardiac Arrhythmias: Update on Mechanisms and Clinical Managements

**DOI:** 10.1155/2016/8023723

**Published:** 2016-07-31

**Authors:** Yi-Gang Li, David G. Benditt, Thomas Klingenheben, Kai Hu, Dali Feng

**Affiliations:** ^1^Department of Cardiology, Xinhua Hospital, Shanghai Jiao Tong University School of Medicine, Shanghai 200092, China; ^2^The Cardiac Arrhythmia Center, University of Minnesota Medical School, Minneapolis, MN 55812, USA; ^3^Goethe University Frankfurt, Frankfurt, Germany; ^4^Private Practice, Bonn, Germany; ^5^Department of Internal Medicine I, University of Würzburg, Würzburg, Germany; ^6^Metropolitan Heart and Vascular Institute, Minneapolis, MN 55433, USA

Atrial fibrillation (AF) is one of the most common arrhythmias in adults and is associated with a high incidence of stroke and heart failure (HF). Despite the advance of AF catheter ablation during the past decades, the high reoccurrence rate of AF after catheter ablation urges improvements of diagnostic approaches, therapies, and technologies. P. D. Dallaglio et al. reviewed the role of adenosine in pulmonary vein isolation in a meta-analysis of 11 studies. The analysis revealed that adenosine is useful to unmask dormant connection (DC) after a first ablation procedure and further ablation at sites of DC would reduce the rate of redo procedures for postablation AF recurrence. The authors also suggested that the use of adenosine should be accompanied by sufficient waiting time.

Cryoablation is an equivalent alternative to radiofrequency ablation for paroxysmal atrial fibrillation. According to the work of S. Conti et al., the use of second-generation cryoballoon was associated with lower procedure duration and fluoroscopy time and comparable procedural success as compared to first-generation cryoballoon.

Cardioversion is used widely in AF patients. The researches of Y. M. Rochlani et al. and D. Wang et al. showed the safety and efficiency of cardioversion for AF patients. Y. M. Rochlani and colleagues found that external electrical cardioversion during an AF related hospitalization could significantly reduce in-hospital stroke, mortality, length of stay, and cost for hospitalization. D. Wang et al. revealed that cardioversion during AF catheter ablation does not impair the maintenance of sinus rate or the recovery of cardiac function.

Even though digoxin has been used in clinical practice to treat heart diseases for decades, the proof of benefit is scarce. The new meta-analysis by S. Chamaria et al. confirms recent evidence that the use of digoxin increases all-cause mortality in all atrial fibrillation patients. In contrast, digoxin is not associated with higher mortality in the important subgroup of patients with AF and HF.

Reactive oxygen species (ROS) are well known to play a role in ischemic heart diseases, whereas their significance in arrhythmias is less well established. A. A. Sovari reviewed several possible ways for ROS to induce arrhythmia including causing focal activity and reentry, altering multiple cardiac ionic currents, promoting cardiac fibrosis, and impairing gap junction function.

Malignant ventricular arrhythmia is always on the top of the list of heart diseases. There is a continuous need of research with regard to revealing the pathogenetic mechanisms and identifying new targets of treatment. Activation of sympathetic nerves after myocardial infarction plays an important role in sudden cardiac death and left ventricular remodeling. C.-Y. Li and Y.-G. Li reviewed the sympathetic rejuvenation after MI. Degeneration and death of sympathetic fibers occur within and around the infarction zones. After MI, sympathetic fibers would regenerate back to the myocytes around the infarcted area. The authors point out that, as part of sympathetic remodeling after MI, excessive sympathetic nerve sprouting might be a potential mechanism for fatal arrhythmia in chronic MI.

Most of heart diseases have multiple causes of which genetics represent a major determinant. Along with the rapid development of gene sequencing and genome-wide association study, single nucleotide polymorphism (SNP) is associated with detection of high-risk population, diagnosis of diseases, and sensitivity to targeted treatment. The study of F. Galati et al. suggests that RyR2 QQ2958 genotype might identify a subgroup of ICD implanted patients at particular high risk of malignant ventricular arrhythmias.

Commotio Cordis is defined as the mechanical stimulation of the heart which induces ventricular fibrillation and sudden cardiac death. It happens mainly in sports. D. H. Wolbrom et al. reviewed the mechanisms and clinical management of ventricular arrhythmias under this situation. In the acute event, rapid defibrillation with AED could save life; however, catheter ablation might be the option for the few patients who develop ventricular arrhythmias during chronic stage. The mechanism of Commotio Cordis is still unclear. In an experimental swine model of chest blow trauma, C. Madias et al. found that stretch activation of the L-type calcium channel and intracellular calcium overload do not seem to play a key role in preventing ventricular arrhythmias.

Skin burns at the site of an indifferent electrode patch are a rare complication after radiofrequency catheter ablation. In a study of H. Ibrahim et al., the incidence of significant skin burns was 0.28%. Higher BMI, procedure time, and postprocedure pain were the factors predicting skin burn.

Historically, a prolonged PR interval by itself does not necessarily imply clinical consequences. Interestingly, using data from a large population-based study, M. P. Husby et al. found that a long PR interval was associated with higher LV mass, LV stroke volume, and LV end-systolic and end-diastolic volumes. Increased ventricular volume and wall stress might lead to arrhythmias.

We hope you enjoy this issue which succinctly explores the depth and breadth of currently available clinical management and mechanisms of atrial fibrillation and ventricular arrhythmias.

*Yi-Gang Li* *David G. Benditt* *Thomas Klingenheben* *Kai Hu* *Dali Feng*

